# A Preliminary Study of Bacterioplankton Community Structure in the Taiyangshan Wetland in Ningxia and Its Driving Factors

**DOI:** 10.3390/ijerph191912224

**Published:** 2022-09-27

**Authors:** Rui-Zhi Zhao, Wei-Jiang Zhang, Wen Zhang, Zeng-Feng Zhao, Xiao-Cong Qiu

**Affiliations:** 1 School of Civil and Hydraulic Engineering, Ningxia University, Yinchuan 750021, China; 2 Ningxia Supervision Institute for Veterinary Drugs and Animal Feedstuffs, Yinchuan 750004, China; 3 School of Life Science, Ningxia University, Yinchuan 750021, China

**Keywords:** Taiyangshan wetland in Ningxia, bacterioplankton community structure, co-occurrence pattern network, driving factors

## Abstract

The Taiyangshan Wetland, a valuable wetland resource in the arid zone of central Ningxia, is critical for flood storage and drought resistance, climate regulation, and biodiversity protection. Nevertheless, the community structure and diversity of bacterioplankton in the Taiyangshan Wetland remains unclear. High-throughput sequencing was used to analyze the differences in bacterioplankton structure and major determinants in the Taiyangshan Wetland from April to October 2020. The composition and diversity of the bacterioplankton community varied significantly in different sampling periods but showed negligible differences across lake regions. Meanwhile, the relative abundances of bacterioplankton Bacteroidetes, Actinobacteria, Firmicutes, Chloroflexi, Tenericutes, Epsilonbacteraeota, and Patescibacteria were significantly different in different sampling periods, while the relative abundances of Cyanobacteria in different lake regions were quite different. Network analysis revealed that the topological attributes of co-occurrence pattern networks of bacterioplankton were high, and bacterioplankton community compositions were complicated in the month of July. A mantel test revealed that the bacterioplankton community in the entire wetland was affected by water temperature, electrical conductivity, dissolved oxygen, salinity, total nitrogen, ammonia nitrogen, chemical oxygen demand, fluoride, and sulfate. The bacterioplankton community structure was affected by ten environmental parameters (e.g., water temperature, dissolved oxygen, salinity, and permanganate index) in April, while the bacterioplankton community was only related to 1~2 environmental parameters in July and October. The bacterioplankton community structure in Lake Region IV was related to seven environmental parameters, including dissolved oxygen, pH, total nitrogen, and chemical oxygen demand, whereas the bacterioplankton community structures in the other three lake regions were related to two environmental parameters. This study facilitates the understanding of the bacterioplankton community in wetlands in arid areas and provides references to the evaluation of aquatic ecological management of the Taiyangshan Wetland.

## 1. Introduction

Wetland plays a pivotal role in hydrological and biogeochemical cycles, biodiversity protection, culture, and tourism as a transition zone between terrestrial and aquatic systems [1,2]. Complex biological composition and high species diversity are the characteristics of an aquatic ecosystem where bacterioplankton plays an essential part. It is primarily driven by the material cycle of most elements and is the key to a deep understanding of the structure and function of the aquatic ecosystem [3,4,5]. The change in the bacterioplankton community structure is closely related to the water environment and has an early warning effect on potential environmental threats. The diversity of bacterioplankton can be used to judge the health status of an aquatic ecosystem [4,6,7], where its functional diversity also determines the function of an aquatic ecosystem to a certain extent [8]. Therefore, exploring the species composition of bacterioplankton, the spatiotemporal dynamic changes in community structure, and its relationship with environmental factors are essential to studying the aquatic ecosystem.

Recently, high-throughput sequencing technology has provided a new, cost-effective means for bacterial community analysis, completely changing the understanding of the bacterial community [9,10,11,12]. The revolutionary development of sequencing technology can provide more data to reveal the bacterial composition in environmental samples and re-examine the ecological role of bacterioplankton in the geographical distribution of aquatic ecosystems such as wetlands and lakes [13,14,15].

The Ningxia Hui Autonomous Region is located in the northwest of inland China, typically the mid-latitude region of the northern hemisphere. The temperate continent has an arid and semi-arid climate. Located in the basin of the Kushui River in the arid area in central Ningxia, the Taiyangshan Wetland was identified as a national wetland park in 2018, with geographical coordinates of 106°32′01″~106°40′58″ E and 37°23′59″~37°29′17″ N. It is windy and rainy all year round, with strong evaporation. The total area of the Taiyangshan national wetland park is 2447.50 hm^2^, the wetland area is 1492.7 hm^2^, and the water area is 654.8 hm^2^. The terrain gradually rises from north to south, with altitudes ranging from 1330 to 1345 m. The wetland water area comprises permanent freshwater lakes, seasonal saltwater lakes, and inland salt marshes. The Taiyangshan Wetland is located at the intersection of many important ecological functional areas, including the hilly and gully soil and water conservation ecological function area of the Loess Plateau, the desertification prevention and ecological protection and control area of Ningxia, and the arid area in central Ningxia ecological improvement area. It is critical for flood storage and drought resistance, regulating microclimate, providing waterfowl breeding grounds, and protecting biodiversity in the central arid zone. It is a rare and unique wetland in the arid area of central Ningxia. The level of eutrophication in wetlands has increased, and bio-diversity has gradually decreased as a result of the dramatic impact of human activities and excessive utilization of water resources on the water area and water quality of wetland. Therefore, there is an urgent need to comprehensively evaluate the ecological status of Taiyangshan Wetland and promote wetland ecological management and ecological remediation. Understanding the wetland bacterioplankton community structure and its dynamic changes is important for systematically evaluating the aquatic ecosystem. However, the bacterioplankton community structure of the Taiyangshan Wetland and its driving factors remain unclear.

The bacterioplankton community structures of the Taiyangshan wetland were examined in three sampling periods using high-throughput sequencing technology. This study has three objectives: (1) analyze the composition of the bacterioplankton community structure within wetlands and how it has changed over time; (2) compare the pattern of the bacterioplankton community between different sampling intervals and lake areas; and (3) identify the main environmental parameters that influence the composition of the bacterial community. The findings of this study will provide crucial information for assessing and managing the aquatic ecosystem of the Taiyangshan Wetland, as well as a scientific foundation for the preservation and effective management of wetland water ecological remediation and biodiversity.

## 2. Materials and Methods

### 2.1. Target Area and Distribution of Sampling Sites

**Figure 1:**
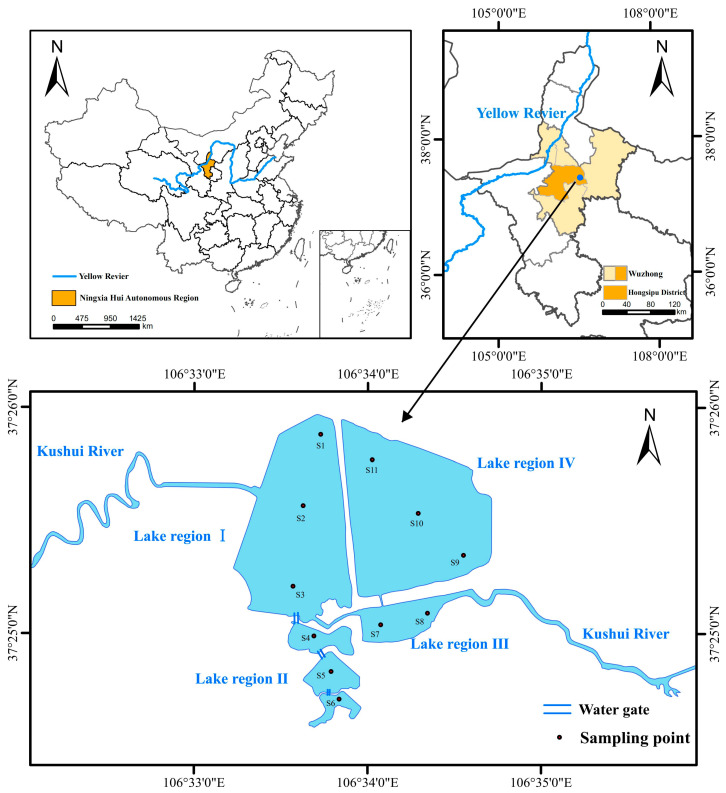
Distribution of sampling sites in the Taiyangshan wetland.

The Taiyangshan Wetland water area is divided into four lake regions based on the actual geographical pattern and water area characteristics, with 11 sampling sites (S1~S11, Figure 1). Lake Region Ⅲ connects the water source of the Kushui River to Lake Regions Ⅰ and IV. Lake Region II is a freshwater spring lake formed by the natural upwelling of underground carbonate fissure karst water. Two perennial sluice gates were used to connect the three flowing water bodies in Lake Region II, which then flowed into Lake Region Ⅰ via another sluice gate. All incoming water was discharged into the lower river channel through the drain of Lake Region Ⅰ. Lake Region III was also connected to Lake Region I and Lake Region IV through a sluice. There is no connection between Lake Region Ⅰ and Lake Region IV and between Lake Region II and Lake Region Ⅲ (see Figure 1). The sampling time is April, July, and October 2020.

### 2.2. Sample Collection and Measurement of Physical and Chemical Indicators

Each sample (4 L) was mixed with surface water (at a depth of 0.5 m) and deep water (at a depth of 2.0 m) in equal amounts. After labeling, the water sample was placed in a polyethylene sampling bottle and stored in an incubator below 4 °C before being transported to the laboratory. The water samples for DNA analysis were filtered through 0.22 μm filter membrane within 24 h (under sterile conditions). The filter membranes were stored at −80 °C until DNA extraction. The sample pretreatment process was carried out in a sterile environment to avoid contamination of samples by external bacteria.

Water temperature (WT), electrical conductivity (Cond), salinity (Sal), dissolved oxygen (DO), pH, total dissolved solids (TDS), and chloridion (Cl^−^) were all measured using a YSI Pro-plus portable water quality analyzer. Fluoride (F^−^) was measured on-site using a HACH HQ40d portable water quality analyzer. Chlorophyll a (Chl *a*) was measured on-site by HACH Hydrolab DS5X. Total nitrogen (TN), total phosphorus (TP), available phosphorus (AP), ammonia nitrogen (NH_4_^+^-N), permanganate index (COD_Mn_), and chemical oxygen demand (COD_Cr_) were determined according to the method provided by the *Water and Wastewater Detection and Analysis Method (4th edition) (Ministry of Ecology and Environment of the People’s Republic of China 2002)*. Sulfate (SO_4_^2−^) was tested according to *the water quality determination of the sulfate-Gravimetric method (GB 11899-89)*.

### 2.3. Extraction, High-Throughput Sequencing, and Bioinformatics Analysis of DNA

Total DNA from all filters was extracted using NucleoSpin 96 soi (MACHEREY-NAGEL, Germany) following the manufacturer’s protocol. 338F (5′-ACTCCTACGGGAGGCAGCA-3′) and 806R (5′-GGACTACHVGGGTWTCTAAT-3’) were used as primer sets to 16S rDNA (V3 + V4) region for PCR amplification [16,17,18]. Thermal cycling consisted of the following conditions: initial denaturation at 95 °C for 5 min; then 25 cycles of denaturation at 95 °C for 30 s, annealing at 50 °C for 30 s, and extension at 72 °C for 40 s; with final extension at 72 °C for 7 min. Successful PCR amplification was verified by 2% agarose gel electrophoresis. The amplification products were mixed at equimolar ratios, purifying using a Monarch DNA gel extraction kit (New England Biolabs, MA, USA), and used for high-throughput sequencing [19,20]. Sequencing was performed by Biomarker Co., Ltd. (Beijing, China) using the Illumina MiSeq 2500 platform with the PE250 strategy.

The paired reads were joined using FLASH (version 1.2.11) [21], then the joined reads were processed using QIIME 1.9.1 [22]. Before primer sequences were detected and trimmed by Cutadapt (version 1.9.1) [23], the raw sequences were denoised, sorted, and separated using Trimmomatic (version 0.33) [24]. After filtering chimeras UCHIME (version 8.1) [25], the remaining sequences were clustered into OTUs (>97% sequence similarity) using USEARCH (version 10.0) [26], OTUs were filtered using a threshold of 0.005% of all sequence counts [27]. The taxonomic identity of representative sequences for each OTU was determined using the Silva reference database (Release 132, http://www.arb-silva.de, (accessed on 20 March 2021)) [28]. The α diversity was calculated using Mothur (version 1.3.0) [29].

### 2.4. Statistical Analysis

In this study, 16 physical and chemical parameters related to the water environment are measured, and Kolmogorov–Smirnov normal distribution test is used to determine whether each water environment variable conforms to normal distribution. The results demonstrated that most water environment parameters did not comply with a normal distribution (*p* < 0.05). Due to the non-normal distribution of the data, the Dunn test is used for multiple comparisons between various sampling periods and lake areas, whereas the Kruskal–Wallis test is used for difference analysis. The Dunn test results are corrected by the Bonferroni method, and this process is completed in R by the R package “rstatix” (version 0.7) [30]. The nonmetric multidimensional scaling (NMDS) ranking based on Bray–Curtis distance is used to explore the differences in the Taiyangshan Wetland bacterioplankton community structure. The R-value obtained from the Analysis of Similarity (ANOSIM) is used to quantify the degree of difference within the bacterioplankton community. The larger the R-value, the higher the degree of difference between groups. This analysis is completed using the R “vegan” package (version 2.6-2) [31] in R.

This study reveals the co-occurrence pattern of bacterioplankton via network analysis. In order to better display the results of network analysis, the low abundance and low-frequency groups (relative abundance less than 0.005 at least 5 samplings) are filtered in advance, and the paired Spearman correlation at the genus level is calculated using R”Hmisc” package [32]. The correlation coefficient matrix is formed using the correlation coefficient ≥ |0.7| with a *p*-value less than 0.05 (Benjamini and Hochberg adjusted) as the standard. The co-occurrence pattern network of bacterioplankton is constructed, and calculated network topological attributes, which include node number (nodes), edges number (edges), average weighted degree (AWD), average path length (APL), average clustering coefficient (ACC), diameter, average degree (AD), and density. These parameters reflect the complexity of the composition of the bacterioplankton community to a certain extent. The co-occurrence pattern network is visualized using Gephi (version 0.9.2) [33].

A mantel test is used to determine the primary driving factors of the bacterioplankton community. In this study, the mantel test function in the R”linkET” package (version 0.0.3.5.) [34,35] was used to calculate the correlation between each water environment variable and the relative abundance of bacterioplankton in the Taiyangshan Wetland, which was then combined with the Pearson correlation matrix constructed by the water environment variable to visualize the relationship between the composition of the bacterioplankton community and the water environment variable.

## 3. Results

### 3.1. Characteristics of Water Environment in the Taiyangshan Wetland

Table 1 lists 16 water environment parameters of the Taiyangshan Wetland. The Taiyangshan Wetland water environment was characterized by weak alkalinity, high salt, and fluorine. Among the three sampling periods, WT, pH, NH_4_^+^-N, AP, COD_Cr_, F^−^, and Chl *a* were significantly different (*p* < 0.05). Nevertheless, according to the Kruskal–Wallis test, no significant differences existed in other parameters. In April, the contents of AP and Chl *a* were higher, and COD_Cr_ was much higher than in other months. In October, fluoride content was maximized.

In addition to COD_Cr_, NH_4_^+^-N, and chlorophyll a, the Kruskal–Wallis test revealed that there are also significant differences in water environment parameters such as electrical conductivity, salinity, TDS, TN COD_Mn_, Cl^−^, and SO_4_^2−^ among lake regions (*p* < 0.05). Electrical conductivity, salinity, and TDS in Lake Region IV are much higher than in other lake regions (Supplementary Table S1).

### 3.2. Variations in Bacterioplankton Diversity in Different Sampling Periods and Lake Regions

The bacterioplankton community structure in the Taiyangshan Wetland was measured using Illumina sequencing technology in April, July, and October 2020. After quality control and filtration of 33 bacterioplankton samples, 20,900 OTUs were obtained, with each sample yielding an average of 633 OTUs (mean = 633 ± 193). The sequencing amount can cover the species in the sample according to the species accumulation curve, ACE, Chao 1, and Coverage (Supplementary Figure S1; Table S2).

**Figure 2:**
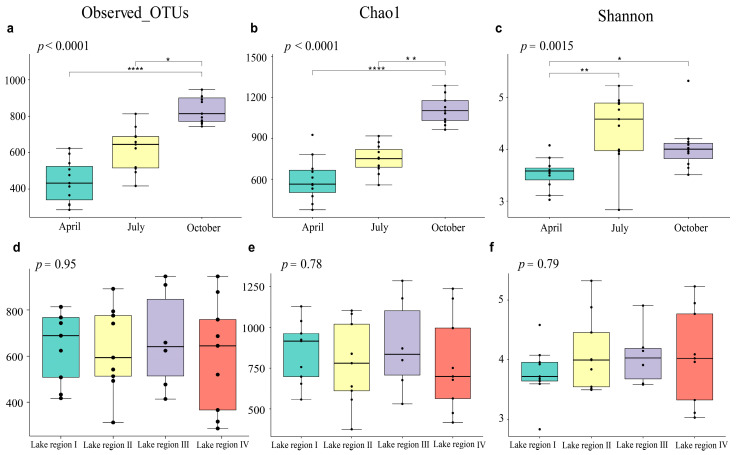
Comparison of alpha diversity indexes between sampling periods and lake regions (Kruskal–Wallis test and Dunn test; *, *p* < 0.05; **, *p* < 0.01; ****, *p* < 0.0001). The variations and differences of Observed_OTUs, Chao1, and Shannon among the three sampling periods were displayed in (**a**–**c**). Following that, they were compared and dispersed across the four lake regions in (**d**–**f**).

Kruskal–Wallis test results showed that Observed_OTUs, Chao1 Index, and the Shannon Index of three sampling periods are significantly different (Observed_OTUs, *p* < 0.0001; Chao1, *p* < 0.0001; Shannon, *p* = 0.0015). Observed_OTUs and Chao1 index were the highest in October, Shannon Index was the highest in July, and the three indexes were the lowest in April. Furthermore, Dunn-test (Bonferroni correction) results showed that each diversity indicator’s difference was not the same among the sampling periods. Observed_OTUs and Chao1 in October were significantly higher than in April and July. The Shannon Index was significantly higher in October than in April, but not significantly different from July (Figure 2a–c).

Observed_OTUs and Chao1 in the four lake regions were in descending order: Lake Region Ⅲ, Lake RegionⅠ, Lake Region II, and Lake Region IV, and Shannon was in the descending order as Lake Region II, Lake Region Ⅲ, Lake Region IV, and Lake Region Ⅰ. The indexes were not significantly different among the four lake regions (Observed OTUs, *p* = 0.95; Chao1, *p* = 0.78; Shannon, *p* = 0.79), as shown in Figure 2d–f. 

### 3.3. Community Structures of Bacterioplankton in Different Sampling Periods and Lake Regions

**Figure 3:**
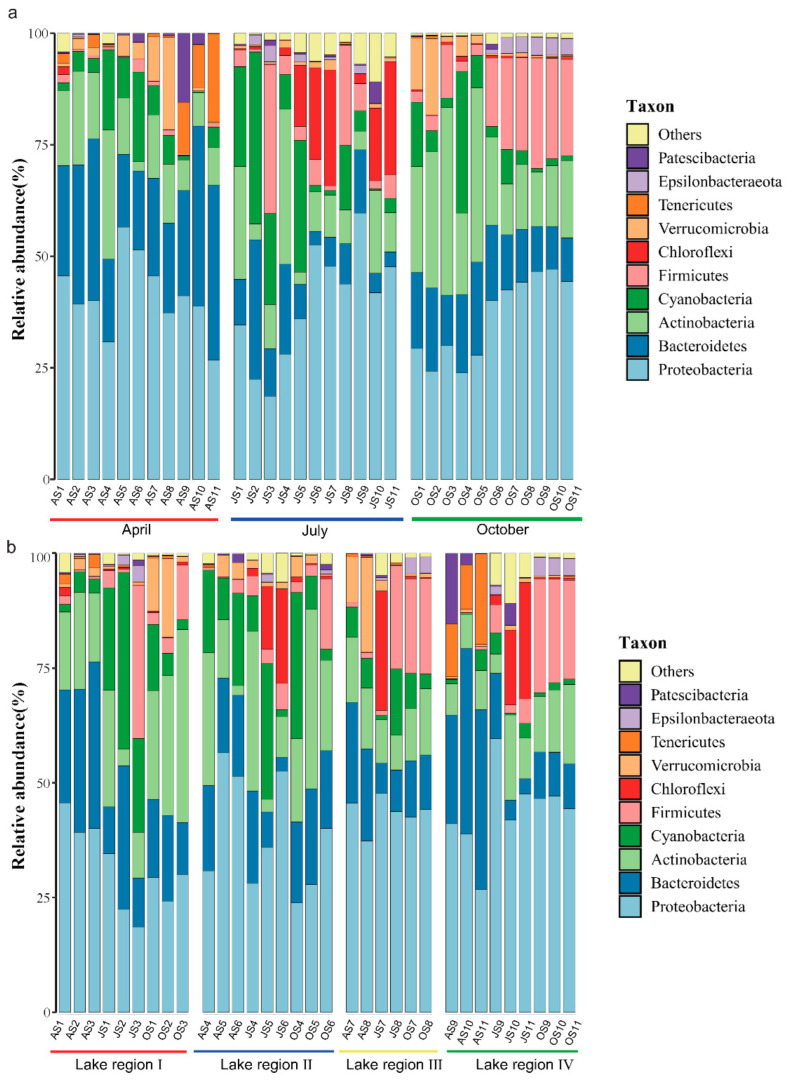
Relative abundances of the dominant bacterial phyla in sampling periods (**a**) and lake regions (**b**). Phyla with relative abundance <0.5% were defined as “other”.

The Taiyangshan Wetland bacterioplankton community is composed of 34 phyla, and the top ten phyla of the whole community’s relative abundance are Proteobacteria (38.96%), Bacteroidetes (17.17%), Actinobacteria (15.85%), Cyanobacteria (8.97%), Firmicutes (7.42%), Chloroflexi (3.44%), Verrucomicrobia (2.86%), Tenericutes (1.53%), Epsilonbacteraeota (0.95%), and Patescibacteria (0.94%), as shown in Figure 3. The results of the Kruskal–Wallis test and Dunn’s test showed significant differences in most bacterioplankton phyla during the sampling period, except for Proteobacteria, Cyanobacteria, and Verrucomicrobia (*p* < 0.05, Supplementary Figure S2a) However, only Cyanobacteria significantly differed among different lake areas (*p* < 0.05, Supplementary Figure S2b). Regarding the family level, considerable differences in the bacterioplankton community structure have been observed between each sample (Figure S3). The overwhelming seasonal pattern was also reflected at this level, especially between Spring and October. Seasonal transitions have been observed between families within the same phylum. For instance, Burkholderiaceae of the Proteobacteria phylum were enriched in spring, while Enterobacteriaceae peaked in Autumn (Figure S3a). Although the bacterioplankton community structure varies strongly with sampling periods, the relative abundances of Burkholderiaceae and Microbacteriaceae still differ among the four lake regions (Figure S3b).

**Figure 4:**
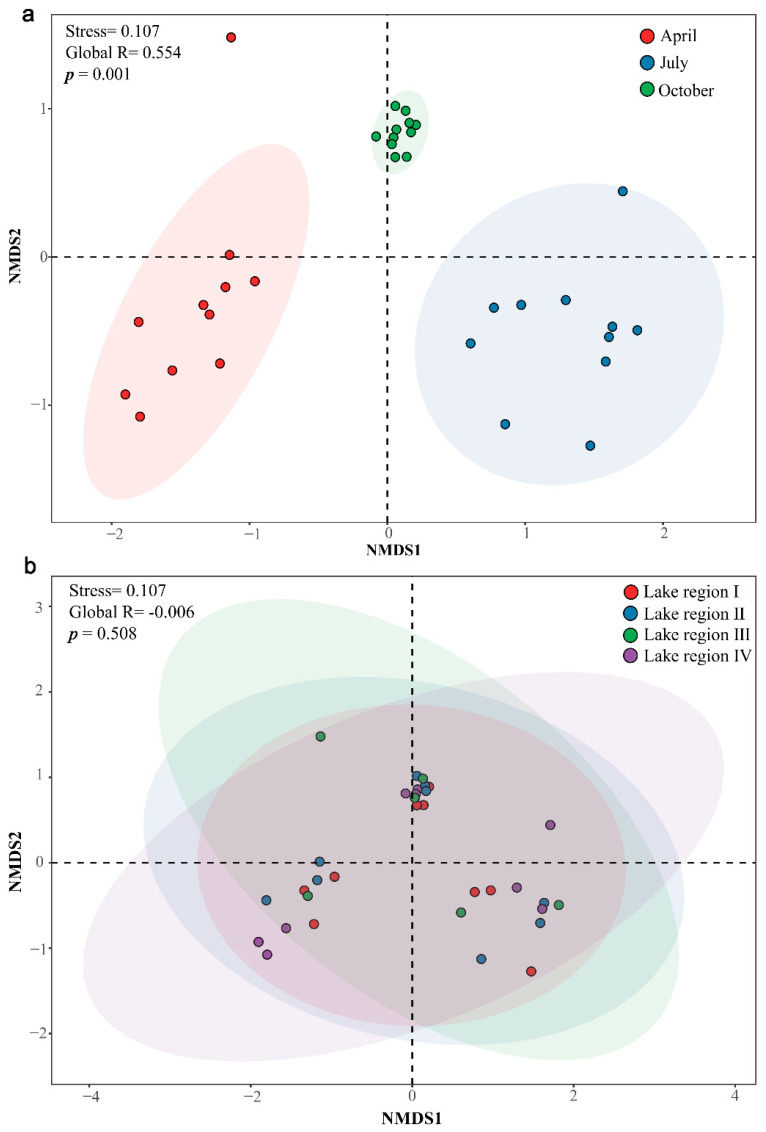
NMDS (Nonmetric multidimensional scaling analysis) and ANOSIM for bacterioplankton communities in sampling periods (**a**) and lake regions (**b**) on Bray–Curtis similarity. Stress < 0.2 provides a good representation in NMDS. Global R > 0 means the grouping is valid. Moreover, p < 0.05 indicates a significant difference. Ellipses are 95% confidence intervals around the centroid. Ellipses are 95% confidence intervals around the centroid. The bacterioplankton community structure varied among sampling periods (**a**), while there were no differences among lake regions (**b**).

The NMDS with Bray–Curtis distance was used to assess the similarity of bacterioplankton communities across sampling periods and lake areas. ANOSIM statistical test showed that the composition of the bacterioplankton community of Taiyangshan Wetland differs significantly between sampling periods (Global R = 0.554, *p* = 0.001, Figure 4a), but no significant difference was observed between different lake areas (Global R = −0.006, *p* = 0.508, Figure 4b). The impact of the sampling period on the composition of the bacterioplankton community structure is much higher than that of geospatial space (Global R, 0.554 vs. −0.006, Figure 4).

**Figure 5:**
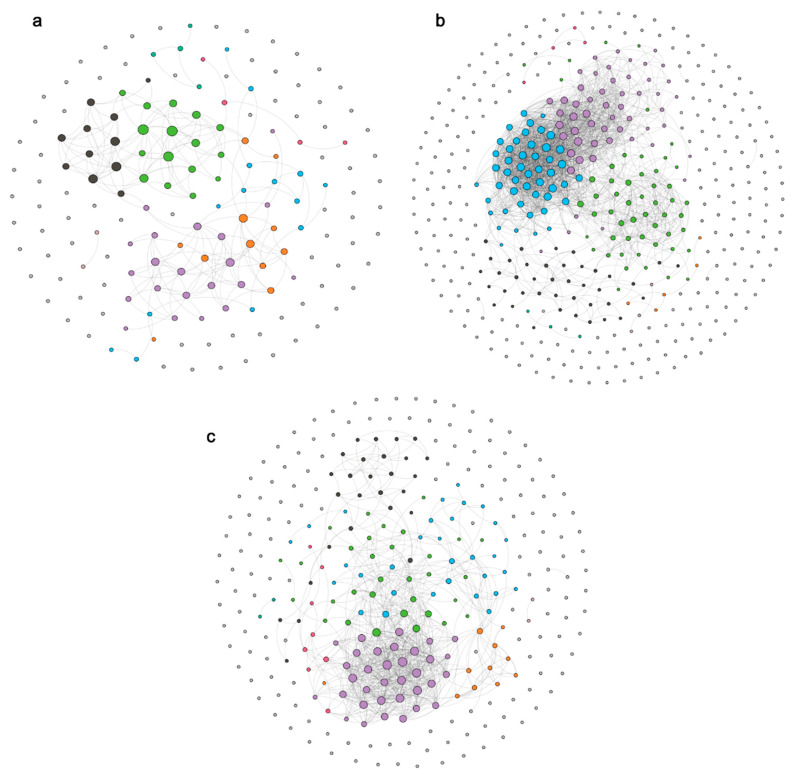
Co-occurrence networks of bacterioplankton in different sampling periods: (**a**) April, (**b**) July, (**c**) October.

The three bacterioplankton co-occurrence pattern networks were constructed based on the bacterioplankton community structure of each sampling period to investigate the complexity of the composition of the Taiyangshan Wetland bacterioplankton community. In April, the co-occurrence pattern networks of bacterioplankton comprised 166 nodes connected by 212 edges, AWD was 1.118, ACC was 0.102, the diameter was 5, network density was 0.008, and APL was 1.822. In July, the co-occurrence pattern networks were composed of 440 nodes connected by 2006 edges, AWD was 3.916, ACC was 0.123, the diameter was 8, network density was 0.01, and APL was 2.387. In October, the co-occurrence pattern networks were composed of 302 nodes connected by 792 edges, AWD was 2.264, ACC was 2.264, the diameter was 6, network density was 0.009, and APL was 2.274 (Figure 5, Supplementary Table S3). The results show that the second co-occurrence pattern network is larger and more complex than the other network structures, implying that the complexity of the composition of the bacterioplankton community is the highest in July.

### 3.4. Correlation Analysis between Bacterioplankton Community Structure and Water Environment Variables

**Figure 6:**
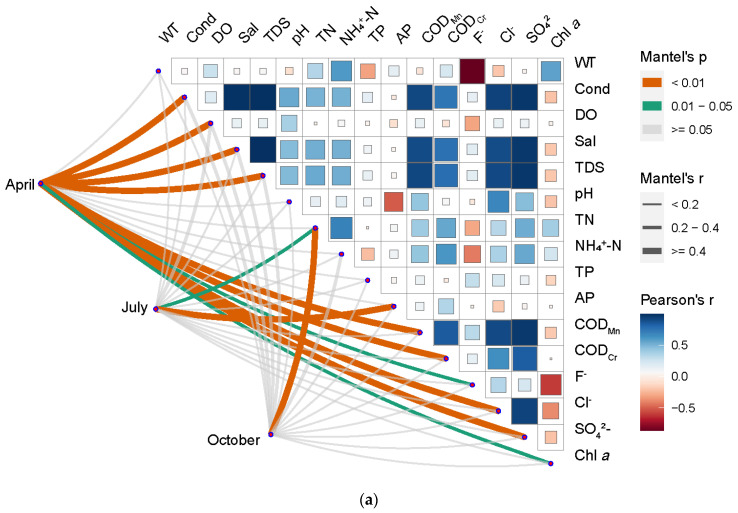

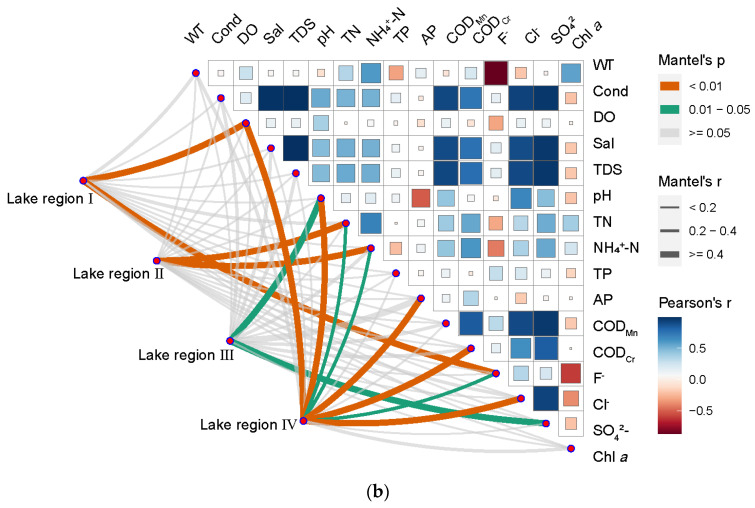
Environmental drivers of bacterioplankton community in the Taiyangshan wetland evaluated by mantel tests: Sampling periods (**a**), lake regions (**b**). Pairwise comparisons of water parameters are shown with a color gradient denoting Pearson’s correlation coefficient. The edge width represents the corresponding distance correlations (Mantel’s r), and the edge color denotes the statistical significance (Mantel’s p).

A Mantel test was used to explore which water environment parameters are significantly related to the composition of the bacterioplankton community. As shown in Figure 6a, in April, electrical conductivity (r = 0.62, *p* = 0.001), dissolved oxygen (r = 0.44, *p* = 0.001), salinity (r = 0.63, *p* = 0.001),TDS (r = 0.64, *p* = 0.001), COD_Mn_ (r = 0.58, *p* = 0.004), COD_Cr_ (r = 0.56, *p* = 0.002), F^−^ (r = 0.62, *p* = 0.001), Cl^−^ (r = 0.68, *p* = 0.025), SO_4_^2−^ (r = 0.68, *p* = 0.001), and Chl *a* (r = 0.24, *p* = 0.045) were found to be significantly correlated with the bacterioplankton community structure in the Taiyangshan Wetland. In July, TN (r = 0.30, *p* = 0.024) and TP (r = 0.66, *p* = 0.001) were significantly correlated with the bacterioplankton community. However, only TN was significantly correlated with the bacterioplankton community structure in October (r = 0.45, *p* = 0.009).

Dissolved oxygen (r = 0.55, *p* = 0.008) and F^−^ (r = 0.55, *p* = 0.001) have a significant impact on the bacterioplankton community in Lake Region I. TN (r = 0.60, *p* = 0.005) and NH_4_^+^-N (r = 0.68, *p* = 0.006) are significantly correlated with bacterioplankton community of Lake Region II. The bacterioplankton community of Lake Region III is significantly correlated with pH (r = 0.55, *p* = 0.025) and SO_4_^2−^ (r = 0.50, *p* = 0.032). In Lake Region IV, dissolved oxygen (r = 0.55, *p* = 0.025), pH (r = 0.55, *p* = 0.025), TN (r = 0.55, *p* = 0.025), NH_4_^+^-N (r = 0.55, *p* = 0.025), AP (r = 0.55, *p* = 0.025), COD_Cr_ (r = 0.55, *p* = 0.025), F^−^ (r = 0.55, *p* = 0.025), and Cl^−^ (r = 0.55, *p* = 0.025) have a strong effect on bacterioplankton community (see Figure 6b).

## 4. Discussion

In this study, 33 water samples were collected from the Taiyangshan Wetland during three sampling periods. The composition and diversity of the bacterioplankton community were analyzed to determine the relationship between the bacterioplankton community and environmental parameters. A preliminary understanding of the wetland bacterioplankton community structure was obtained, which helps monitor water quality and evaluate and manage the Taiyangshan wetland.

The Taiyangshan Wetland is located in the arid area of central Ningxia, primarily formed by the Kushui River and groundwater supply. The Kushui River is the second first-class tributary of the Yellow River in Ningxia. It has a very high background mineralization degree and total hardness, with maximum values of 21,400 mg/L and 6488 mg/L, respectively. The farmland recession in the whole basin is large, and the water quality is poor. Our results show that under the direct influence of the background water quality, evapotranspiration, and lake structure of the Kushui River, the environmental parameters (e.g., salinity, TDS, COD_Mn_, and COD_Cr_) of Lake Region IV are much higher than those of other lake regions, which has become the primary source of water environment characteristic differences among the four lake regions. Because the nitrogen and phosphorus content in Lake Region Ⅲ is at a high level, it provides good conditions for algae growth where the content of chlorophyll a is high [36,37]. The water from the Kushui River does not enter Lake Region II. Therefore, except for fluoride and chlorophyll a, the water environment variable in Lake Region II is lower than in other lake regions. As shown in Figure 6, there is a strong correlation between fluoride and chlorophyll a and water temperature among the water environment parameters in the wetland, and there is also a significant correlation between Ph and available phosphorus. The differences in available phosphorus, fluoride, and chlorophyll between various sampling periods are caused by the possible differences in water temperature and pH. However, the excessively high variable detection value in Lake Region IV in April is the fundamental reason for the significant difference in COD.

In recent years, many studies using high-throughput sequencing technology have shown that the bacterioplankton community in the dilute aquatic ecosystem has high diversity [38,39,40]. Observed_OTUs represent the intuitive number of OTUs in the study, while the Chao1 Index and Shannon Index estimate the richness and diversity of the bacterioplankton community. In the Taiyangshan Wetland, the abundance order of bacterioplankton is ranked as October > July > April, while diversity is ranked as July > October > April. Different sampling periods significantly impact the diversity of bacterioplankton, similar to many reported results [41,42,43]. However, there is no statistically significant difference among the four lake regions regarding Observed_OTUs, Chao1 Index, and Shannon Index. The research on the composition of the bacterioplankton community in the Taiyangshan Wetland found that the sum of relative abundance of Proteobacteria, Bacteroidetes, Actinobacteria, Cyanobacteria, Firmicutes, Chloroflexi, Verrucomicrobia, Tenericutes, Epsilonbacteraeota, Patescibacteria exceeds 98%, showing the richness of the composition of the community. The most dominant group is Proteobacteria, followed by Bacteroidetes and Actinobacteria. Researchers have shown that the top three groups in the lake are Proteobacteria, Actinobacteria, and Bacteroidetes [44,45]. Actinobacteria and Bacteroidetes have strong distance attenuation patterns within the scope of large-scale research, whereas Proteobacteria is easier to diffuse through water flow, air, and waterfowl migration [46,47,48,49]. This study investigates the spatiotemporal pattern of the bacterioplankton community in the Taiyangshan Wetland. According to the NMDS results in Figure 4, the composition of the bacterioplankton community is significantly different according to the sampling period. Indeed, some environmental parameters (e.g., water temperature, pH) are quite different in the three sampling periods (Table 1). Water temperature and pH affect other environmental parameters (Figure 6), and their significant seasonal changes can strongly affect the composition of the bacterioplankton community [50]. As shown in Supplementary Figure S2, there are substantial differences in the relative abundance of the seven main phyla of the bacterioplankton community. Furthermore, the degree of separation of the bacterioplankton community between different lake areas is low compared to the sampling period due to the small geographical scale difference in the lake area. Thus, the bacterioplankton community differs only slightly between lake areas. We found that Cyanobacteria in its bacterioplankton community is significantly lower than in other lake areas due to special water quality conditions and the lake structure of Lake Region IV (see Figure S2).

Network topological parameters are the direct embodiment of the cohesion and connectivity of the network. These parameters can be used to evaluate the complexity of the co-occurrence pattern networks [51]. Our study constructed the co-occurrence pattern networks of bacterioplankton between different sampling periods. A comparison of the topological parameters of the three networks revealed that the number of nodes and edges in the co-occurrence pattern networks was maximized in July, suggesting that the bacterioplankton community has a high contact frequency. Meanwhile, the higher network density and clustering coefficient also indicate that the connectivity and complexity of the bacterioplankton co-occurrence pattern networks are higher in July, indicating that the complexity of the bacterioplankton community is lower in April and October than that in July. Furthermore, modularity is a closely connected network area, which can usually represent niche overlap or species aggregation [52]. Higher bio-diversity can promote the interaction between bacterial communities, thus reducing network modularity [53]. In the Taiyangshan Wetland, the Shannon Index of the bacterioplankton community was maximized and minimized in July and April, respectively. As a result, the degree of modularity of the co-occurrence pattern networks of the bacterioplankton community in July is lower than that in April.

Currently, it is considered that the bacterial community in a static water lake is primarily influenced by internal environmental factors (species interaction). The bacterial community structure is thought to be the result of a random distribution of external environmental factors and different habitats in flowing water areas [54]. The water area of the Taiyangshan Wetland is a flowing water area, and multiple external environmental factors may influence the bacterioplankton community structure [55,56]. Overall, the bacterioplankton community in the whole wetland is affected by water temperature, electrical conductivity, DO, salinity, TN, NH_4_^+^-N, COD_Cr_, F^−^, and SO_4_^2−^ (Supplementary Figure S4). The research shows that salinity and temperature are the critical factors in developing the microbe community structure in different environments [57,58]. Salinity shapes the microbial community distribution pattern and is stronger than the temperature effect [59]. Nonetheless, the strong influence of salinity and temperature on the bacterioplankton community does not mean that the effects of other environmental factors on the bacterioplankton community can be ignored. It has been demonstrated that dissolved oxygen significantly impacts the abundance of bacterioplankton [60]. Our study also obtained similar results. Dissolved oxygen is significantly correlated with the bacterioplankton community of the whole Taiyangshan Wetland (*p* < 0.01, r = 0.26, Supplementary Figure S4). Moreover, the bacterioplankton community of Lake Regions Ⅰ and IV significantly correlated with the dissolved oxygen in April. Notably, the nutrient levels represented in this study by total nitrogen and ammonia nitrogen are also closely related to bacterioplankton. The influence of nutrients on community structure is attributed to the circulation of nutrients by the bacterioplankton community. The change in its concentration directly affects bacterial nutrition metabolism and community development [61,62]. Chemical oxygen demand is a comprehensive index used to characterize water pollution caused by organic matter [63]. The bacterioplankton community structure varies in waters depending on chemical oxygen demand levels [64]. The results of this study show that organic pollution is relatively common in the Taiyangshan Wetland, and there was a significant correlation between the bacterioplankton community and COD_Cr_, particularly in April and Lake Region IV. The bacterioplankton community in this study is not significantly influenced by pH, even though pH should be one of the major environmental factors determining the structure of the bacterioplankton community [65].

## 5. Conclusions

The first step in monitoring environmental and ecological conditions is to understand the structural and environmental driving factors of the bacterioplankton community. The present research shows that the bacterioplankton community in the Taiyangshan Wetland comprised 34 phyla of which Proteobacteria, Bacteroidetes, Actinobacteria, Cyanobacteria, Firmicutes, Chloroflexi, Verrucomicrobia, Tenericutes, Epsilonbacteraeota, and Patescibacteria were the main species. The bacterioplankton community structure changes significantly with the sampling period, but the difference between lake areas is insignificant. When the topological parameters of three sampling periods were compared, it was found that the bacterioplankton co-occurrence pattern networks have higher connectivity and cohesion in July, indicating that the bacterioplankton community structure is more complex. Water temperature, electrical conductivity, dissolved oxygen, salinity, TN, NH_4_^+^-N, COD_Cr_, F^−^, and SO_4_^2−^ were environmental parameters that influence the overall structure of the wetland bacterioplankton community. The driving factors were different between different sampling periods and lake areas. This study conducted a preliminary investigation into the bacterioplankton community structure in the Taiyangshan Wetland and its driving factors, but the precise effects of each environmental variable on the bacterioplankton community structure have not been established, and it should be encouraged in future research. Furthermore, the assembly mechanism of the bacterioplankton community in the river lake continuum generated by the Taiyangshan Wetland and the Kushui River should be studied to improve water ecological monitoring and management of the basin of the Kushui River.

## Figures and Tables

**Table 1:** Water environment parameters (mean ± SD) of the Taiyangshan wetland in different sampling periods.

Environmental Parameters	April	July	October	Statistical Significance
WT (°C)	19.78 ± 1.54	25.25 ± 2.63	11.15 ± 1.49	*
Cond (μS/cm)	15,372 ± 12,827	18,946 ± 16,102	17,406 ± 15,074	ns
DO (mg/L)	5.34 ± 1.11	6.51 ± 1.63	5.52 ± 0.97	ns
Sal (ppt)	10.93 ± 10.5	11.67 ± 10.36	11.13 ± 10.77	ns
TDS (mg/L)	11,584 ± 10,165	12,185 ± 10,320	11,404 ± 9946	ns
pH	7.68 ± 0.17	8.65 ± 0.70	8.56 ± 0.57	*
TN (mg/L)	2.94 ± 1.89	2.77 ± 1.82	1.65 ± 1.66	ns
NH_4_^+^-N (mg/L)	1.34 ± 0.91	1.68 ± 1.59	0.25 ± 0.1	*
TP (mg/L)	0.04 ± 0.02	0.04 ± 0.01	0.08 ± 0.07	ns
AP (mg/L)	0.019 ± 0.012	0.003 ± 0.002	0.002 ± 0.001	*
COD_Mn_ (mg/L)	6.58 ± 3.87	5.11 ± 3.03	6.25 ± 4.33	ns
COD_Cr_ (mg/L)	160.35 ± 208.24	35.48 ± 27.38	28.04 ± 20.3	*
F^−^ (mg/L)	3.99 ± 0.39	2.98 ± 0.27	4.95 ± 0.4	*
Cl^−^ (mg/L)	8388 ± 8144	12,236 ± 12,783	16,707 ± 15,374	ns
SO_4_^2−^ (mg/L)	6135 ± 5695	5624.14 ± 5239	5835 ± 5626	ns
Chl *a* (mg/L)	27.46 ± 17.64	25.1 ± 10.19	11.19 ± 8.04	*

ns, non-significant; *, *p* < 0.05. The means of each water environment parameter were the averaged value over all lake regions.

## Data Availability

The data are not publicly available due to privacy.

## Supplementary Materials

The following supporting information can be downloaded at: https://www.mdpi.com/article/10.3390/ijerph191912224/s1, Table S1: Water environment parameters (mean ± SD) of the Taiyangshan wetland in different lake regions; Table S2: Alpha diversity indexes of each sampling sites; Table S3: The topology parameters of co-occurrence patterns network in different sampling periods; Figure S1: Species accumulation curves of bacterioplankton communities in the Taiyangshan wetland; Figure S2: Relative abundance of different phyla between sampling periods (a) and lake regions (b) (* *p* < 0.05, *** *p* < 0.001, Kruskal-Wallis test); Figure S3: Relative abundances of the dominant bacterial families in sampling periods (a) and lake regions (b). Family with relative abundance <1% were defined as “other”; Figure S4: Environmental drivers of bacterioplankton community in the Taiyangshan wetland evaluated by mantel tests. Pairwise comparisons of water parameters are shown with a color gradient denoting Pearson’s correlation coefficient. The edge width represents the corresponding distance correlations (Mantel’s r), and the edge color denotes the statistical significance (Mantel’s p).
