# Supplementary material for: A Preliminary Study of Bacterioplankton Community Structure in the Taiyangshan Wetland in Ningxia and Its Driving Factors
Source: Int J Environ Res Public Health. 2022 Sep 27;19(19):12224. doi: 10.3390/ijerph191912224 (PMC9565018; doi:10.3390/ijerph191912224)
Supplement: Supplementary file 1 [file ijerph-19-12224-s001.zip › ijerph-1899169-supplementary.pdf]

Supplementary Material

Table S1. Water environment parameters (mean ± SD) of the Taiyangshan wetland in different lake regions.

Environmental Parameters	Lake Region I	Lake Region II	Lake Region III	Lake Region IV	Statistical Significance
WT (°C)	18.92 ± 7.92	17.96 ± 4.65	19.08 ± 5.23	19.08 ± 7.14	ns
Cond (μS/cm)	11154 ± 1320	5717 ± 1078	10593 ± 1957	39284 ± 6818	*
DO (mg/L)	6.15 ± 1.65	5.44 ± 1.22	5.46 ± 0.87	5.99 ± 1.44	ns
Sal (ppt)	6.33 ± 1.20	3.63 ± 1.00	6.43 ± 1.32	26.99 ± 5.02	*
TDS (mg/L)	7307 ± 1077	4019 ± 756	7319 ± 1404	26784 ± 5002	*
pH	8.42 ± 0.84	7.79 ± 0.22	8.25 ± 0.33	8.71 ± 0.72	ns
TN (mg/L)	1.25 ± 0.58	0.94 ± 0.83	5.26 ± 1.11	3.3 ± 0.82	*
NH_4^+-N (mg/L)	0.60 ± 0.21	0.43 ± 0.4	1.29 ± 1.11	2.1 ± 1.67	*
TP (mg/L)	0.06 ± 0.04	0.03 ± 0.01	0.07 ± 0.08	0.05 ± 0.03	ns
AP (mg/L)	0.014 ± 0.016	0.005 ± 0.005	0.005 ± 0.004	0.007 ± 0.008	ns
COD_{Mn} (mg/L)	4.92 ± 0.59	2.72 ± 0.95	4.11 ± 0.92	11.55 ± 1.66	*
COD_{Cr} (mg/L)	29.3 ± 8.36	13.97 ± 6.79	39.25 ± 27.31	204.19 ± 209.76	*
F^- (mg/L)	3.79 ± 0.87	4.03 ± 0.81	3.51 ± 0.78	4.42 ± 0.97	ns
Cl^- (mg/L)	8292 ± 4484	2802 ± 682	5703 ± 1842	30729 ± 8564	*
SO_4^{2-} (mg/L)	3247 ± 161	1662 ± 201	3332 ± 382	14374 ± 773	*
Chl *a* (mg/L)	14.68 ± 8.68	24.1 ± 14.12	39.57 ± 14.15	12.77 ± 4.64	*

ns, non-significant; *, $p < 0.05$. The means of each water environment parameter were the averaged value over all three sampling periods.

Table S2. Alpha diversity indexes of each sampling site.

Sample ID	OTU	ACE	Chao1	Simpson	Shannon
Apr.S1	508	623.0323	654.6538	0.0397	4.077
Apr.S2	433	555.4241	558.1017	0.0516	3.5934
Apr.S3	624	1058.43	924.9157	0.0483	3.6792
Apr.S4	312	356.5453	375.3704	0.0585	3.4958
Apr.S5	594	821.7487	781.0312	0.0684	3.5451
Apr.S6	542	582.4524	612.0227	0.0561	3.8382
Apr.S7	477	708.4499	677	0.0443	3.6002
Apr.S8	414	528.8062	531.1852	0.0664	3.5822
Apr.S9	316	877.1245	564.3333	0.0719	3.1085
Apr.S10	286	426.2628	417.3182	0.0547	3.3232
Apr.S11	366	494.2923	475.1695	0.0926	3.0277
Jul.S1	813	911.8909	916.6	0.0247	4.584
Jul.S2	417	847.7398	697.9149	0.1523	2.832
Jul.S3	689	747.8786	757.2432	0.0617	3.9569
Jul.S4	493	666.3244	638.087	0.0512	3.9942
Jul.S5	742	835.1615	838.8	0.0877	4.4584
Jul.S6	513	542.1076	557.4516	0.0149	4.8798
Jul.S7	659	931.5553	871.8983	0.0244	4.9072
Jul.S8	624	819.3439	799.375	0.0503	3.9121
Jul.S9	687	730.8191	751.1875	0.0102	5.2281
Jul.S10	520	803.8534	679.0128	0.0169	4.7677
Jul.S11	645	690.3387	699.1852	0.0142	4.9475
Oct.S1	744	987.2557	963.3782	0.0592	3.7176
Oct.S2	814	1097.0193	1128.6875	0.0664	3.6428

Oct.S3	768	1084.9856	1039.527	0.0881	3.9256
Oct.S4	794	1103.4786	1102.431	0.0445	4.0049
Oct.S5	776	988.3786	1020.8627	0.1059	3.5115
Oct.S6	892	1039.5933	1083.2059	0.0112	5.3239
Oct.S7	946	1204.4478	1177.9942	0.0521	4.1481
Oct.S8	909	1245.9388	1285.9106	0.0515	4.2055
Oct.S9	759	1035.7322	996.4264	0.0668	3.9634
Oct.S10	878	1199.3923	1177.0197	0.0577	4.0909
Oct.S11	946	1249.4291	1237.2338	0.0614	4.0235

Table S3. The topology parameters of co-occurrence pattern networks in different sampling periods.

	Nodes	Edges	AWD	Diameter	Density	Modularity	ACC	APL
April	166	212	1.118	5	0.008	0.628	0.102	1.822
July	440	2006	3.916	8	0.01	0.421	0.123	2.387
October	302	792	2.264	6	0.009	0.448	0.117	2.274

AWD, average weighted degree. ACC, average clustering coefficient. APL, average path length.

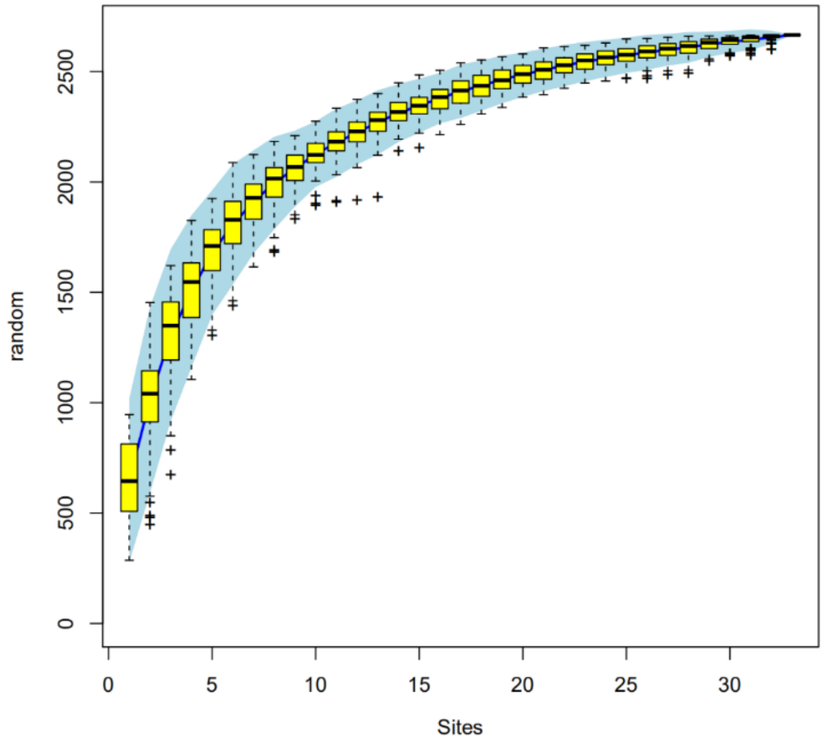

Figure S1. Species accumulation curves of bacterioplankton communities in the Taiyangshan wetland.

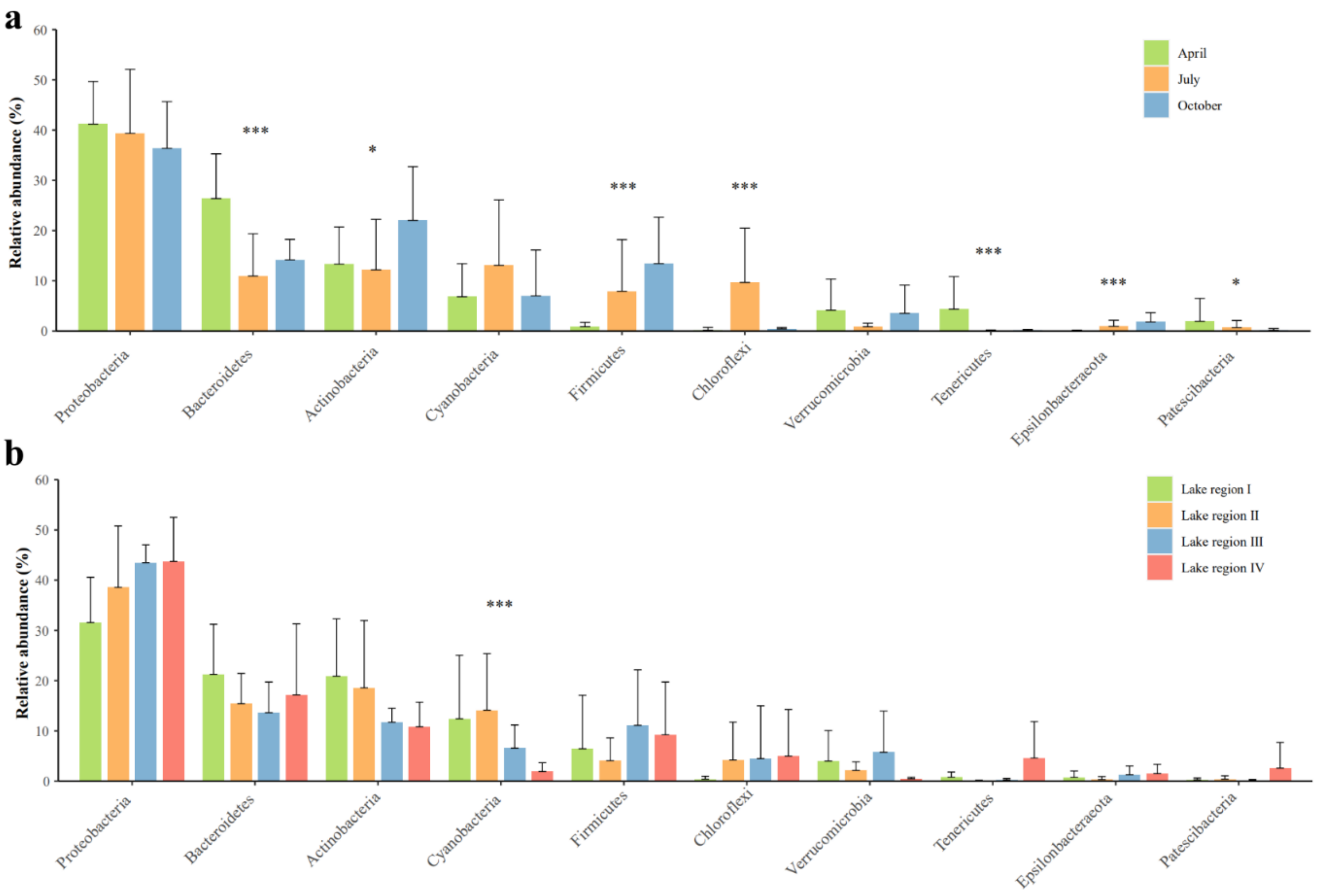

Figure S2. Relative abundance of different phyla between sampling periods (**a**) and lake regions (**b**) (* $p < 0.05$, *** $p < 0.001$, Kruskal–Wallis test).

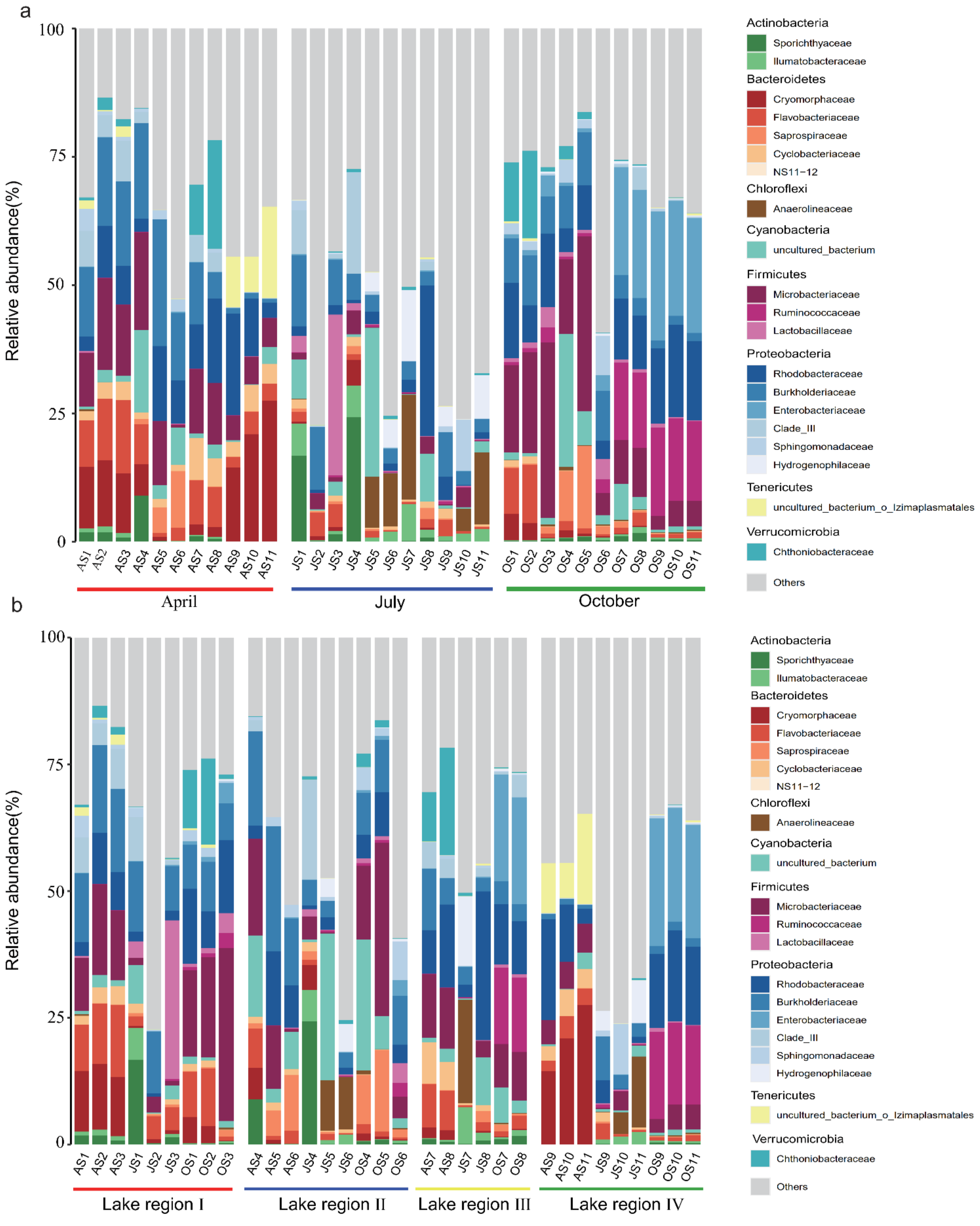

Figure S3. Relative abundances of the dominant bacterial families in sampling periods (**a**) and lake regions (**b**). Family with relative abundance <1% were defined as "other".

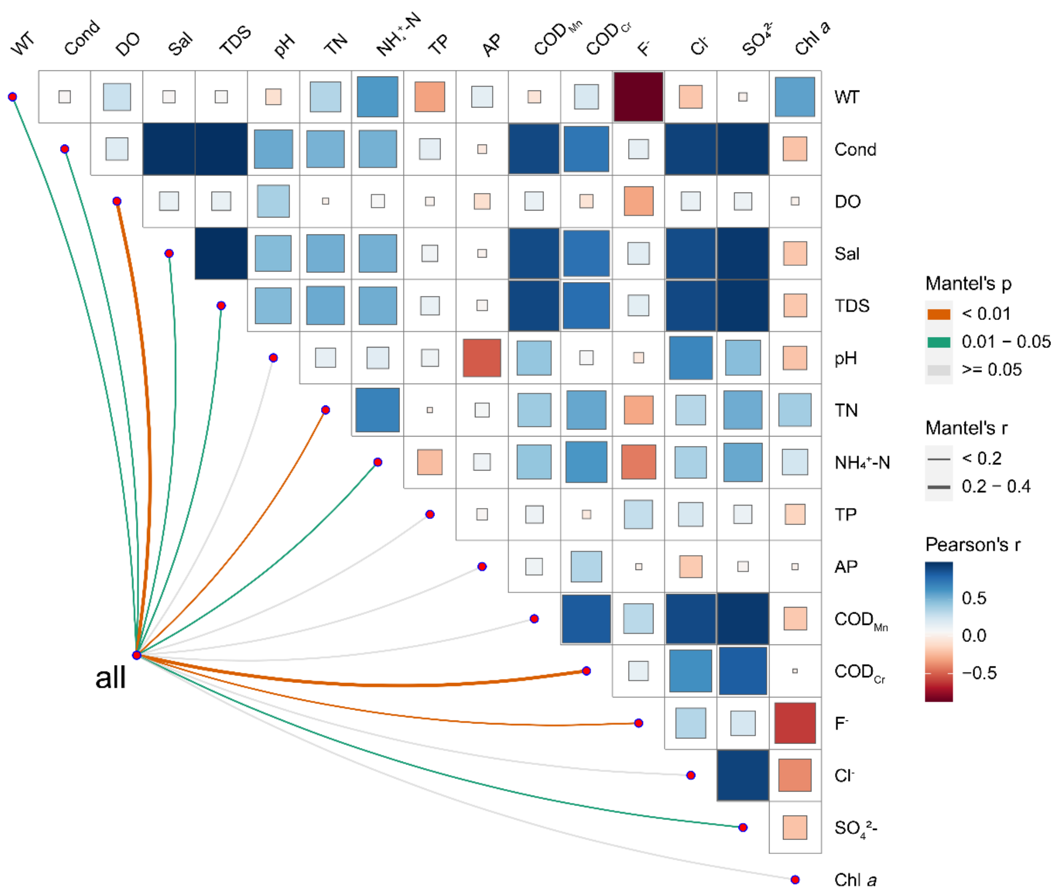

Figure S4. Environmental drivers of bacterioplankton community in the Taiyangshan wetland evaluated by mantel tests. Pairwise comparisons of water parameters are shown with a color gradient denoting Pearson's correlation coefficient. The edge width represents the corresponding distance correlations (Mantel's r), and the edge color denotes the statistical significance (Mantel's p).
